# A novel splicing mutation in the *PRPH2* gene causes autosomal dominant retinitis pigmentosa in a Chinese pedigree

**DOI:** 10.1111/jcmm.14278

**Published:** 2019-03-20

**Authors:** Jingliang Cheng, Jiewen Fu, Qi Zhou, Xiaohong Xiang, Chunli Wei, Lisha Yang, Shangyi Fu, Md. Asaduzzaman Khan, Hongbin Lv, Junjiang Fu

**Affiliations:** ^1^ Key Laboratory of Epigenetics and Oncology, The Research Center for Preclinical Medicine Southwest Medical University Luzhou Sichuan China; ^2^ Institute of Medical Technology Xiangtan Medicine and Health Vocational College Xiangtan Hunan China; ^3^ Department of Ophthalmology Affiliated Hospital of Southwest Medical University Luzhou Sichuan China; ^4^ The Honors College University of Houston Houston Texas; ^5^ Department of Molecular and Human Genetics Baylor College of Medicine Houston Texas

## 1 INTRODUCTION

Retinitis pigmentosa (RP) (OMIM: 268000) is a rare, heterogeneous group of inherited ocular disorders that results in a progressive retinal degeneration.^1^, ^2^ The *PRPH2* gene (NM_000322.4) (OMIM: 179605), also known as *RDS*, *AOFMD*, *AVMD*, *CACD2*, *DS*, *MDBS1*, *PRPH*, *rd2*, *RP7* and *TSPAN22*, is located on chromosome 6p21.1 with three exons spanning 26 395 bp length in human genome (GRCh38/hg38) that encodes a putative protein with 346 amino acids.^3^ The PRPH2 protein (NP_000313.2) is a member of the transmembrane 4 superfamily, also known as the tetraspanin family. The majority of the members are cell‐surface proteins which were identified by the presence of four hydrophobic domains. The PRPH2 protein is a membrane‐associated glycoprotein, which is restricted to the area of photoreceptor outer segment discs.^4^ PRPH2 functions as an adhesion molecule by stabilization and compaction of outer segment discs. PRPH2 and ROM1 (OMIM: 180721) can be assembled into noncovalent tetramers (heterodimer) in vivo using disulphide bonds and higher order disulphide‐linked oligomers, thereby involving in photoreceptor disc morphogenesis.^5^

Mutations in the *PRPH2* gene are involved with assorted blinding diseases of the retina, inducing degenerations in both central retinal and peripheral retinal.^6^, ^7^, ^8^ The relationships between the mutations in the *PRPH2* gene and the resultant diseases in the patients are variable; making genotype/phenotype correlations different. *PRPH2* mutation in RP patients and genotype/phenotype relationship have not been well described in the Chinese population.

## 2 MATERIALS AND METHODS

### 2.1 Ethics statement, proband, pedigree and clinical assessment

**Figure 1:**
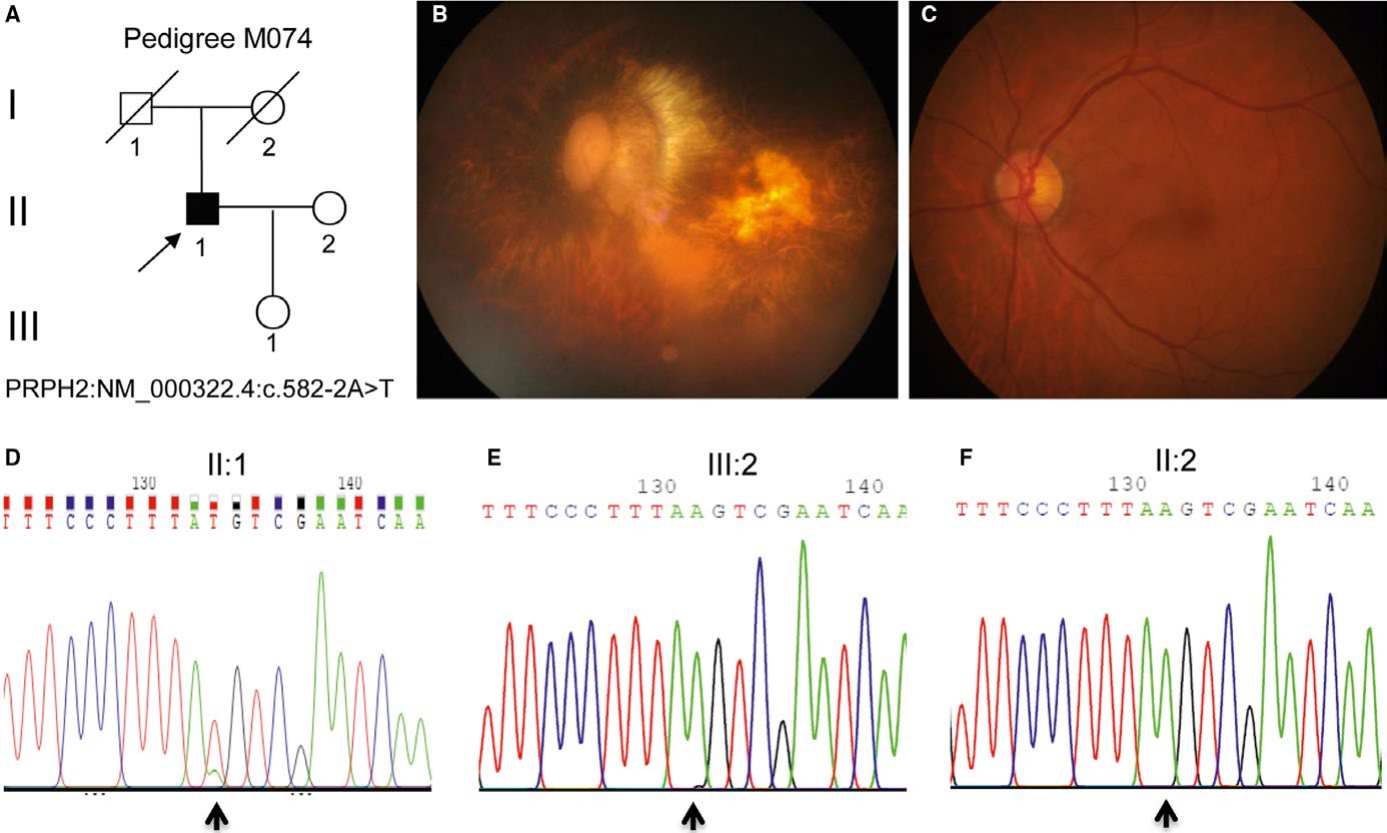
A M074 pedigree with retinitis pigmentosa in the proband and pyrogram profiles for variant verification by Sanger sequencing. A, A M074 pedigree. Family numbers and disease‐causing mutation are presented. Normal individuals are shown as clear circles (females), whereas the affected individual is shown as filled symbol (square). The filled square with an arrow indicates the proband (II: 1) with the splicing variant of the *PRPH2* gene: NM_000322.4:c.582‐2A>T. B, Representative FP of patient II: 1 from left eye. C, FP from the normal matched individual. D‐F, Indicate the sequencing results in II: 1 (heterozygous mutant type), II: 2 (wild‐type) and III: 1 (wild‐type) respectively. The arrows indicate the mutation at the nucleotide position NM_000322.4: c.582‐2A>T in the *PRPH2* gene

The study was approved by the Ethics Committee of *Southwest Medical University*. Written informed consent was obtained from the participants, in accordance with the guidelines of the Declaration of Helsinki (2013 Revision). The pedigree (M074) consisted of a proband (Figure 1A, pedigree II: 1, arrow). For detailed clinical assessments, a clinical history and ophthalmic examination were previously described.^9^, ^10^ Genomic DNA (gDNA) was extracted using a reported phenol/chloroform method.^11^

### 2.2 Target sequencing and data analysis

Targeted next‐generation sequencing (TGS) analyses were performed on the gDNA sample of the proband from family M074.^12^, ^13^ The capture Agilent probes were used as in previously published studies^2^, ^12^, ^13^, ^14^ with a retinal disease capture panel with 195 retinal disease‐causing genes. Library construction and sequencing were used according to the manufacturer's protocols.^15^ Data analysis and sequenced variants identification were described in detail previously.^2^, ^10^

### 2.3 Primer design and PCR amplification

A primer pair M074‐PRPH2 was designed containing the NM_000322.4: c.582‐2A>T in the *PRPH2* gene (M074‐PRPH2‐868L: 5‐ttcagcgcctagaacagtga‐3; M074‐PRPH2‐868R: 5‐tcgaagagccaaatgaggag‐3, 411 bp). For variant validation and pedigree segregation analysis, PCR amplification was used to by using gDNA of all available individuals.^2^ PCR amplification for sequencing was performed in a total volume of 20 µL.

### 2.4 Sanger sequencing and co‐segregation analysis

The amplified PCR products were then Sanger sequenced using a primer M074‐PRPH2‐868L. Co‐segregation analysis in the pedigree was finished by using Sanger sequencing results.

### 2.5 Protein structure prediction and bioinformatics analysis

A search for conserved domains in protein or nucleotide sequence was done through the online program (https://www.ncbi.nlm.nih.gov/Structure/cdd/wrpsb.cgi). The *PRPH2* gene homologs were analysed using previously described HomoloGene system: (https://www.ncbi.nlm.nih.gov/homologene?Db=homologene&Cmd=Retrieve&list_uids=273).

### 2.6 RNA extraction and reverse transcription‐PCR

RNA was extracted from mice according to our previously reported standard protocol.^2^ Semi‐quantitative RT‐PCR was performed with the primer pair RT‐m‐Prph2 (RT‐m‐Prph2‐L: 5‐tcgtcacacttctcgtctgg‐3; RT‐m‐Prph2‐R: 5‐catctgctgcatcgttcagt‐3, 457 bp) targeting the mouse *Prph2* gene; the mouse β‐actin gene was used as an internal control, which was described previously.^2^

## 3 RESULTS

### 3.1 Proband and clinical characteristics

The proband (Figure 1A, II: 1) was a 60‐year‐old Chinese male, and claimed a reduction in his visual acuity and peripheral field loss in the fourth decade of life. Fundus examination revealed refractive medium opacity, fundus blurring, macular degeneration and posterior pole retinal atrophy in both eyes (Figure 1B and C). Yellow‐white deposits varying in shape and size within the macula were also noticed. The retinal pigment epithelium atrophy and small retinal vessels were observed. Electroretinogram (ERG) assessments showed that amplitudes of rod‐isolated responses and amplitudes of cone‐isolated responses were almost extinguished. The proband's parents and his daughter was normal without retinal disease. As a result, the proband was characterized as adult‐onset RP.

### 3.2 TGS results and putative pathogenic mutation screening

By TGS with the proband gDNA (Figure 1A, pedigree II: 1). A heterozygous, splice site mutation (c.582‐2A>T) at the exon 3 boundary in the *PRPH2* gene (NM_000322.4) was revealed, leading to unknown amino acid changes of the PRPH2 protein C‐terminus (NP_000313.2) (Figure 1A, II: 1). These deleterious and pathogenic aspects of the *PRPH2*: c.582‐2A>T variant are listed in Table 1. Thus, this splicing mutation c.582‐2A>T in the *PRPH2* gene most likely damaged the protein function in this Chinese RP pedigree. This variant was demonstrated to be novel by searching in database ExAC (http://exac.broadinstitute.org/gene/ENSG00000112619) and HGMD (http://www.hgmd.cf.ac.uk/ac/gene.php?gene=PRPH2).

**Table 1:** Characteristics of PRPH2 variant and analysis of disease‐causing effects

Gene	Exon	Variation				Disease‐causing	ExAC
		Nucleotide#	Protein#	Type	Status		
PRPH2	3	c.582‐2A>T	NA	Splicing	Heter	Damaged	Novel

All nucleotide and amino acid are abbreviated according to the International Union of Pure and Applied Chemistry (IUPAC).

c: variation at cDNA level; ExAC: Exome Aggregation Consortium; Heter: heterozygote; NA: not available; p: variation at protein level; PRPH2: Homo sapiens peripherin 2.

### 3.3 Variant verification and segregation analysis

Confirmation of the variant and co‐segregation analysis was done by Sanger sequencing (Figure 1). The c.582‐2A>T mutation of the *PRPH2* gene was verified to be heterozygous in the proband (pedigree II: 1; Figure 1D), while we revealed the wild‐type gene in the proband's daughter without RP symptoms till test at the ages of 30 (pedigree III: 1; Figure 1E), and the proband's wife had two copies of the wild‐type allele and a normal phenotype (pedigree II: 2; Figure 1F). Therefore, we validated that the c.582‐2A>T mutation in the *PRPH2* gene is co‐segregated with the RP disease phenotype in these pedigree members. Furthermore, the c.582‐2A>T variant was absent in 100 normal, ethnically matched controls by Sanger sequencing. Comprehensively, this finding shows co‐segregation of the variant in this RP pedigree and pinpoints c.582‐2A>T variant role in pathogenesis of this RP disease. Unfortunately, no DNA samples were available due to the death of proband's parents. The c.582‐2A>T variant of the *PRPH2* gene might be de vivo as no any RP phenotypes were claimed in the proband's parents.

### 3.4 Functional effects of the pathogenic mutation c.582‐2A>T for PRPH2

**Figure 2:**
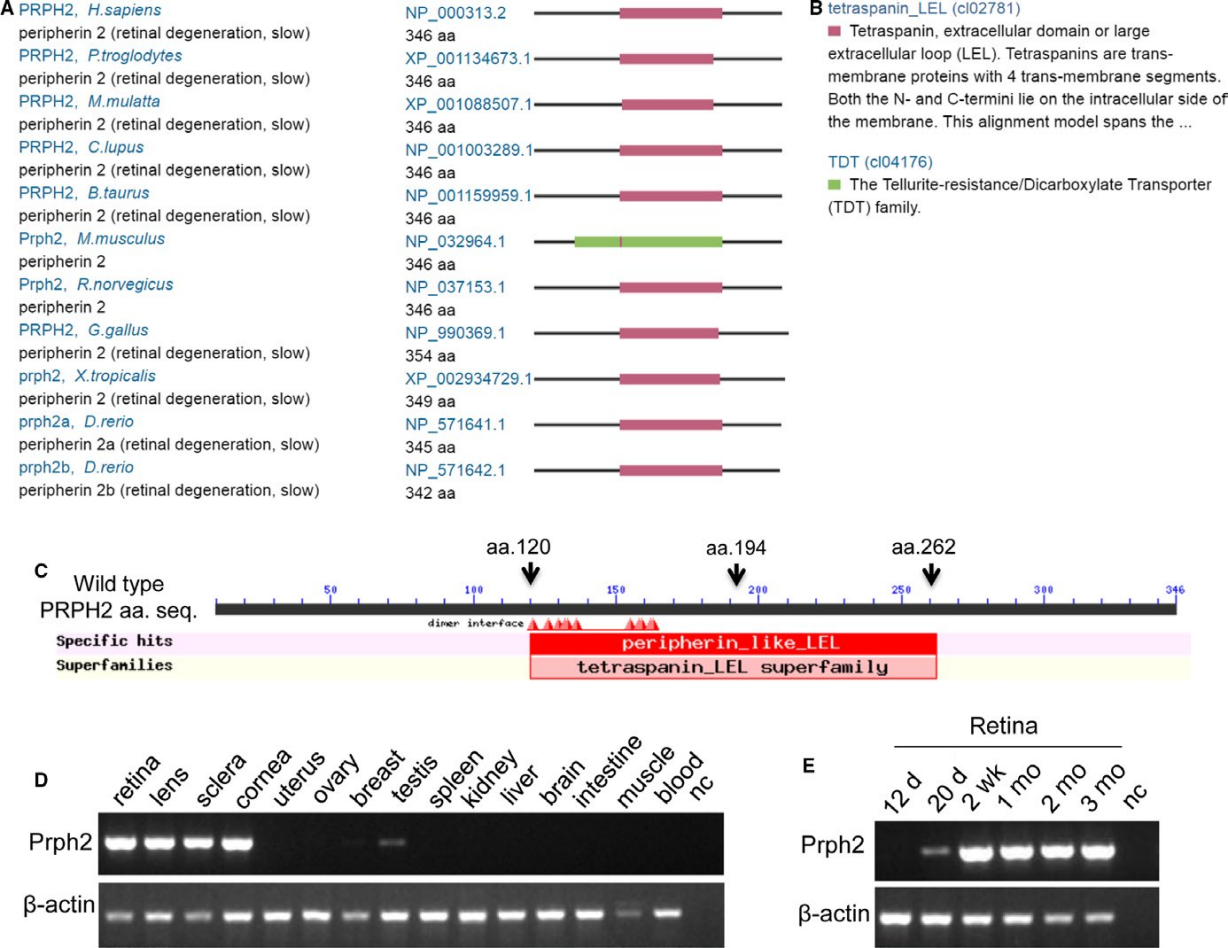
The PRPH2 structure and comparison, and the *Prph2* mRNA expression. A, Orthologous conservation analysis of the PRPH2 in indicated species. B, PRPH2 domains. C, Position of amino acid residues and dimer interface regions from conserved domain. Filled triangles in red indicate dimer interface regions. aa, amino acid. Expression of the *Prph2* mRNA in the indicated tissues (D) and at the indicated development stages or times in retinal tissue (E) in mice. d, day(s); w, week(s); m, month(s); nc, negative control without any template DNA; muscle, skeletal muscle. Whole eye balls from embryos at 12.5 days (12d) and 20.5 days (20d) in panel B respectively

Conserved Domain rpsblast searching found that PRPH2 has two conserved domains (Figure 2A), namely tetraspanin, extracellular domain or large extracellular loop (LEL) (tetraspanin_LEL, cl02781) or the tellurite‐resistance/Dicarboxylate Transporter (TDT) family (TDT, cl04176)) (Figure 2B). The c.582‐2A>T variant is located in the tetraspanin_LEL domain in *H sapiens* (aa.120‐aa.262), leading to amino acid change after 194 (Figure 2C, arrow of wild protein). By orthologous comparison of *H sapiens* PRPH2 to nine other species, including *Pan troglodytes*, *Macaca mulatta*, *Bos taurus*, *Canis lupus*, *Mus musculus*, *Rattus norvegicus*, *Gallus gallus*, *Xenopus tropicalis* and *Danio rerio* (two isoforms), we found that this protein is highly conserved (Figure 2A). Altogether, our investigation revealed that the *PRPH2* heterozygous variant, c.582‐2A>T, might cause adRP disease.

### 3.5 Expression profiles of *Prph2* mRNA level

The *Prph2* expression in the indicated tissues and developmental retinal stages was studied in mice (Figure 2D and E). We found that *Prph2* transcript is highly expressed in the retina, lens, sclera and cornea of the eye; is weakly expressed in the testis; have no detectable expression in the uterus, ovary, breast, spleen, kidney, liver, brain, intestine, skeletal muscle and blood (Figure 2D); and is highly expressed at the latter four different developmental stages of retinal tissue (Figure 2E). The very high expression of *Prph2* in the retinal tissue and ubiquitous expression in other tissues of eyes demonstrated that Prph2 should play an important role in the retinal/eye function.

## 4 DISCUSSION

Earlier diagnosis and managements result in a better prognosis. The *PRPH2* mutation in RP patients and genotype/phenotype relationship with RP have not been well described in the Chinese population. In this study, we have successfully revealed a heterozygous, splicing mutation of the *PRPH2* gene, c.582‐2A>T, in a Chinese pedigree, which led to the adRP disease. The patient's parents did not show any RP phenotypes till death, suggesting that this variant might be de novo. By searching HGMD (access date, September 24, 2018), 125 pathogenic mutations in the *PRPH2* gene have been found, including missenses/nonsenses (83), small deletions (28), small insertions (5), small indels (4), a gross deletion (1) and a gross insertions/duplication (1). To the best of our knowledge, the *PRPH2* mutation c.582‐2A>T is novel, thereby extending mutation spectrums.

The PRPH2 protein belongs to the transmembrane 4 superfamily‐tetraspanin family, which mediates signal transduction events by playing roles in the regulation of cell development, activation, growth and motility. As a membrane‐associated glycoprotein, it was found in the outer segment of rod/cone photoreceptor cells.^4^ PRPH2 functions as an adhesion molecule involving in stabilization and compaction of outer segment discs. PRPH2 and ROM1 (OMIM: 180721) are assembled into noncovalent tetramers (heterodimer) using disulphide bonds and disulphide‐linked oligomers, thus involved in photoreceptor disc morphogenesis.^5^ Prph2 and Rom1 oligomerization are essential for forming photoreceptor outer segment by an intermolecular disulphide bond at Prph2‐C150/Rom1‐C153; disrupting this bond in a C150S‐Prph2 knockin mouse losses complex formation, normal OS structure and function. PRPH2 has the tetraspanin‐LEL domain where it may act as molecular facilitator relating the ability, specific cell‐surface proteins grouping and of signalling complex formation and stability. Variant c.582‐2A>T of PRPH2 is located on the tetraspanin‐LEL domain and near to dimer interface region (Figure 2C), which might affect the formation of outer segment morphogenesis and stability of signalling complexes. Thus, this PRPH2 mutation might affect the formation and heterodimerization, inhibit signalling, thereby we explain the genetic dominance of the PRPH2 mutant allele in our pedigree.

Orthologous comparison of *H sapiens* PRPH2 to nine other species revealed that this protein is highly conserved. Our quantitative RT‐PCR results in mouse showed that *Prph2* mRNA is only highly expressed in the retina, lens and sclera and cornea of the eye, indicating that PRPH2 plays an important role in the retina/eye functions. Comprehensively, our study found that the *PRPH2* heterozygous mutation, c.582‐2A>T, might causes adRP disease.

In conclusion, our research is the first to identify the novel heterozygous mutation c.582‐2A>T of *PRPH2*, which might causes RP disease in our Chinese family, thereby extending mutation spectrums. Our findings can also help in further understanding of adRP molecular pathogenesis, and assist the diagnosis and genetic counselling of the RP disease.

## CONFLICT OF INTEREST

The authors declare no conflict of interest.

## AUTHORS’ CONTRIBUTIONS

JF was in charge of the idea, project design and concept of the study; JC, JiF, LY, SF and CW performed DNA extraction, PCR amplification, sequencing and data analysis; HL, QZ and XX recruited the clinical patients and were in charge of the clinical assessments; JF, JC, SF and MK wrote the manuscript; JF revised the manuscript.

## References

[1] Ali MU , Rahman M , Cao J , Yuan PX . Genetic characterization and disease mechanism of retinitis pigmentosa; current scenario. 3 Biotech. 2017;7:251. 10.1007/s13205-017-0878-3 PMC5515732 28721681

[2] Fu J , Ma L , Cheng J , et al. A novel, homozygous nonsense variant of the CDHR1 gene in a Chinese family causes autosomal recessive retinal dystrophy by NGS‐based genetic diagnosis. J Cell Mol Med. 2018;22:5662‐5669. 30160356 10.1111/jcmm.13841 PMC6201214

[3] Travis GH , Christerson L , Danielson PE , et al. The human retinal degeneration slow (RDS) gene: chromosome assignment and structure of the mRNA. Genomics. 1991;10:733‐739. 1679750 10.1016/0888-7543(91)90457-p

[4] Travis GH , Sutcliffe JG , Bok D . The retinal degeneration slow (rds) gene product is a photoreceptor disc membrane‐associated glycoprotein. Neuron. 1991;6:61‐70. 1986774 10.1016/0896-6273(91)90122-g

[5] Loewen CJ , Moritz OL , Molday RS . Molecular characterization of peripherin‐2 and rom‐1 mutants responsible for digenic retinitis pigmentosa. J Biol Chem. 2001;276:22388‐22396. 11297544 10.1074/jbc.M011710200

[6] Kajiwara K , Hahn LB , Mukai S , Travis GH , Berson EL , Dryja TP . Mutations in the human retinal degeneration slow gene in autosomal dominant retinitis pigmentosa. Nature. 1991;354:480‐483. 1684223 10.1038/354480a0

[7] Wells J , Wroblewski J , Keen J , et al. Mutations in the human retinal degeneration slow (RDS) gene can cause either retinitis pigmentosa or macular dystrophy. Nat Genet. 1993;3:213‐218. 8485576 10.1038/ng0393-213

[8] Farrar GJ , Kenna P , Jordan SA , et al. A three‐base‐pair deletion in the peripherin‐RDS gene in one form of retinitis pigmentosa. Nature. 1991;354:478‐480. 1749427 10.1038/354478a0

[9] Huang J , Fu J , Fu S , et al. Diagnostic value of a combination of next‐generation sequencing, chorioretinal imaging and metabolic analysis: lessons from a consanguineous Chinese family with gyrate atrophy of the choroid and retina stemming from a novel OAT variant. Br J Ophthalmol. 2019;103:428‐435. 30366948 10.1136/bjophthalmol-2018-312347

[10] Yang L , Fu J , Cheng J , et al. A novel variant of the FZD4 gene in a Chinese family causes autosomal dominant familial exudative vitreoretinopathy. Cell Physiol Biochem. 2018;51:2445‐2455. 30537745 10.1159/000495901

[11] Fu J , Li L , Lu G . Relationship between microdeletion on Y chromosome and patients with idiopathic azoospermia and severe oligozoospermia in the Chinese. Chinese Med J. 2002;115:72‐75. 11930664

[12] Wang F , Wang H , Tuan HF , et al. Next generation sequencing‐based molecular diagnosis of retinitis pigmentosa: identification of a novel genotype‐phenotype correlation and clinical refinements. Hum Genet. 2014;133:331‐345. 24154662 10.1007/s00439-013-1381-5 PMC3945441

[13] Zhang Q , Xu M , Verriotto JD , et al. Next‐generation sequencing‐based molecular diagnosis of 35 Hispanic retinitis pigmentosa probands. Sci Rep. 2016;6:32792. 27596865 10.1038/srep32792 PMC5011706

[14] Zhu L , Cheng J , Zhou B , et al. Diagnosis for choroideremia in a large Chinese pedigree by next‐generation sequencing (NGS) and non‐invasive prenatal testing (NIPT). Mol Med Rep. 2017;15:1157‐1164. 28098911 10.3892/mmr.2017.6119 PMC5367376

[15] Salvo J , Lyubasyuk V , Xu M , et al. Next‐generation sequencing and novel variant determination in a cohort of 92 familial exudative vitreoretinopathy patients. Invest Ophthalmol Vis Sci. 2015;56:1937‐1946. 25711638 10.1167/iovs.14-16065 PMC4365990

